# A Temporal Validation Study of Diagnostic Prediction Models for the Screening of Elevated Low-Density and Non-High-Density Lipoprotein Cholesterol

**DOI:** 10.3390/jcm14217617

**Published:** 2025-10-27

**Authors:** Wuttipat Kiratipaisarl, Vithawat Surawattanasakul, Wachiranun Sirikul, Phichayut Phinyo

**Affiliations:** 1 Department of Community Medicine, Faculty of Medicine, Chiang Mai University, 110 Intrawarorot Road, Si Phum Subdistrict, Mueang Chiang Mai District, Chiang Mai 50200, Thailand; wuttipat.k@cmu.ac.th (W.K.); wachiranun.sir@cmu.ac.th (W.S.); 2 Environmental Medicine and Occupational Medicine Excellent Center, Faculty of Medicine, Chiang Mai University, Chiang Mai 50200, Thailand; 3 Department of Biomedical Informatics and Clinical Epidemiology, Faculty of Medicine, Chiang Mai University, Chiang Mai 50200, Thailand; phichayut.phinyo@cmu.ac.th

**Keywords:** Anthropometry, bioelectrical impedance, Clinical Prediction Rules, Decision Support Techniques, dyslipidemia, hypercholesterolemia

## Abstract

**Background/Objectives**: Limited accessibility to hypercholesterolemia diagnosis hinders the primary prevention of cardiovascular disease. Therefore, we conducted a prospective, temporal validation study of two diagnostic prediction models, targeting endpoints of elevated low-density lipoprotein cholesterol (LDL-C, ≥160 mg/dL) and non-high-density lipoprotein cholesterol (non-HDL-C, ≥160 mg/dL). **Methods:** We prospectively recruited workers aged 20–40 years from a single-center, university hospital from March to June 2024 (*n* = 1099). We determined two diagnostic endpoints: elevated LDL-C and non-HDL-C. The predicted probabilities were derived from the binary logistic regression based on gender, metabolic age, and diastolic blood pressure. We assessed three prediction performances: discrimination from area under the receiver-operating characteristic curve (AuROC); calibration slope (C-slope) and calibration-in-the-large (CITL) from the calibration plot; clinical net benefit from decision curve analysis. Recalibration was based on C-slope and CITL, with a socioeconomic subgroup fairness assessment of AuROC, C-slope, and CITL. **Results:** From 1099 eligible participants, we identified 135 (12.3%) elevated LDL-C and 251 (22.8%) elevated non-HDL-C cases. The LDL-C model had poor discrimination (AuROC 0.59; 95%-CI, 0.56–0.62), miscalibration (C-slope 0.64; 95%-CI, 0.39–0.88 and CITL −0.14; 95%-CI, −0.27–−0.02), and negligible investigation reduction. The non-HDL-C model had fair discrimination (AuROC 0.67; 95%-CI, 0.64–0.69), miscalibration (C-slope 0.71; 95%-CI, 0.59–0.83 and CITL −0.07; 95%-CI, −0.17–0.03), and 20% investigation reduction at prevalence threshold probability. Updated model fairness improved compared to the original models. **Conclusions:** Temporal validation demonstrated modest replicability for the elevated non-HDL-C model, with a potential limitation in participants with normal BMI but low muscle and high fat mass. Health practitioners may use updated elevated non-HDL-C models as a non-invasive triage strategy in young adults, with threshold probabilities within the positive clinical net benefit ranges. Further external validation studies in a larger and more diverse population are necessary.

## 1. Introduction

Hypercholesterolemia, the medical condition describing elevated serum cholesterol, is potentially responsible for 54% of atherosclerotic cardiovascular disease (ASCVD) cases throughout the human lifespan (adjusted risk ratio, aRR, 0.46; 95%-CI, 0.41–0.52) [1]. Managing hypercholesterolemia in young adults is, thus, considered a major primary prevention for later-life ASCVD [2,3]. However, gaps in case recognition hinder the initiation of primary prevention, illustrated by the latest nationwide study of Thai young adults aged 20–34 years, which estimates the prevalence of hypercholesterolemia at 17%, though only 5.6% of these cases acknowledge hypercholesterolemia as the underlying disease [4]. As a consequence, alternative surveillance strategies should be evaluated to facilitate the case discovery process. One of the non-invasive, alternative options is the implementation of diagnostic prediction models for both individual risk assessments and public authority use in screening programs. Nevertheless, the major obstacles are the challenges in obtaining all the predictors based on the existing prediction models [5,6,7,8,9]. The required predictors usually have either a restricted availability, such as income [5], or limited clinical practicability, such as serum copper and gamma glutamine transferase [6]. This limitation, thus, presents challenges both for assessing replicability and transportability, and for providing practical use. Moreover, the literature focusing on evaluating the replicability of existing models is limited, hindering the certainty in implementing these strategies across the intended population. Our research thereby aims at temporal validation of the previously published two diagnostic prediction models, predicting the endpoints of elevated low-density-lipoprotein cholesterol (LDL-C) and elevated non-high-density-lipoprotein cholesterol (non-HDL-C), while accommodating simple inputs of three predictors: age, gender, and diastolic blood pressure (DBP) [10]. Therefore, our research objectives are to demonstrate the replicability of the performance metrics—discrimination, calibration, and clinical net benefit—and their ranges across both models in a more recently recruited, prospective cohort of participants derived from the same source population in which the prediction models are intended to be implemented.

## 2. Materials and Methods

### 2.1. Population, Participants, and Source of Data

We carried out consecutive prospective participant recruitment from a single-center, tertiary-care university hospital, from 18 March to 12 June 2024. Inclusion criteria encompassed healthcare and non-healthcare workers in a tertiary-care hospital aged 20–40 years. Exclusion criteria consisted of any previous diagnoses made by the physicians in the hospital medical records coded with the International Classification of Diseases, 10th Revision (ICD-10) before the examination date. Participants with medical conditions that may affect serum cholesterol level, including essential or familial hypercholesterolemia (E78), hypothyroidism (E03), anorexia nervosa (F50), nephrotic syndrome (N04), and current pregnancy (Z34) were excluded [11]. Also, exclusion was applied to individuals with previous treatment using common medications that are known to have effects on serum lipid levels; this restriction included statins of any type, ezetimibe, proprotein convertase subtilisin/kexin type 9 (PCSK9) inhibitors, and fibrates. Finally, we excluded participants with any past usage of common medications with side effects related to the serum lipid profile, including thiazide diuretics, glucocorticoids, amiodarone, and cyclosporin [12].

### 2.2. Predictors

The temporal validation follows the previous publication [10], collecting data on two predictors—gender (male or female), metabolic age (years)—for the LDL-C model, and three predictors—gender (male or female), metabolic age (years), and diastolic blood pressure (DBP, mmHg)—for the non-HDL-C model. Moreover, other predictors were also collected and thereby used to contrast between development and validation cohorts to demonstrate temporal variation in the population parameters. These included the following: age (years), calculated by visiting date minus date of birth divided by 365.25; BMI (kg/m^2^), calculated by weight (kg) divided by the square terms of height in meters (m^2^); waist circumference (cm), measured at the umbilicus by a trained nurse practitioner; estimated fat mass (kg), muscle mass (kg), visceral fat index (unit), basal metabolic rate (kcal/day).

The measurement protocol is outlined as follows. Firstly, trained nurses measured systolic blood pressure (SBP, mmHg) and DBP (mmHg) using an A&D TM-2657P machine (A&D Company, Limited, Ann Arbor, MI, USA). Secondly, the Tanita model BC-418 (Tanita Corporation of America, Arlington Heights, IL, USA), consisting of a four-point plantar bioelectrical impedance analysis (BIA), was utilized to examine body composition. The processes involved participants standing barefoot on the pedestal pad of the machine under instruction from trained nurses, who simultaneously input participant age, gender, and height. A pre-specified 0.6 kg deduction was applied to exclude the weight of the participants’ clothes. The machine then analyzed and reported back accordingly with the incorporation of chronological age, gender, BMI, and four-point BIA into insightful parameters related to body composition, including metabolic age, which was originally identified as an important predictor and included in the model derived from the development cohort. The BIA output slip was then retrieved for raw data, input, and cross-checked by two trained nurses into the hospital information system.

The combinations of predictors used in each model were summed up as the linear predictor, in which the coefficient of the logistic regression was multiplied with the values of each predictor, resulting in a logarithmic term of predicted odds. Linear predictors with a value of 0 denoted odds of 1 (50% predicted probability of having the endpoint), whereas a value of less than 0 indicated a <50% predicted probability, and a value of more than 0 indicated a >50% predicted probability, accordingly.

### 2.3. Diagnostic Endpoints

A separate team of biomedical technicians at the central laboratory of the hospital objectively analyzed the blood samples taken from the participant just before the start of the health examination. The Cobas b 101 system (Roche Holding AG, Basel, Switzerland) was used for the total cholesterol and HDL-C quantification. Cobas LDL-Cholesterol Generation 3 (LDLC3) (Roche Holding AG, Basel, Switzerland) was chosen as a direct quantification method for LDL-C. The chosen cut-off points for the classification of individuals with elevated LDL-C and non-HDL-C were specified at ≥160 mg/dL according to the previously published models [10] and clinical guidelines on ASCVD risk factor control recommended by the American Heart Association and the American College of Cardiology (AHA-ACC) in their 2019 statement [3].

### 2.4. Sample Size Considerations

The calculation of the minimum sample size required for the temporal validation of a multivariable prediction model is based on the method developed by Riley et al. (2024) [13]. In the LDL-C model, the pre-specified input consisted of a prevalence of 13.6%, an area under the receiver-operating characteristic curve (AuROC) of 0.639 with a confidence interval (CI) width of 0.1, a calibration slope of 1 with a CI width of 0.25, an expected-to-observed ratio (E:O) of 1 with a CI width of 0.25, and a linear predictor of −2.04 ± 0.41. Consequently, the minimal sample size was 990 for discriminative performance; 14,448 for the calibration slope; and 1566 for the E:O. In the non-HDL-C model, the pre-specified input consisted of a prevalence of 19.7%, an AuROC of 0.721 with a CI width of 0.1, a calibration slope of 1 with a CI width of 0.25, an E:O of 1 with a CI width of 0.25, and a linear predictor of −1.41 ± 0.81. Consequently, the minimum sample sizes were 624 for discriminative performance, 2951 for the calibration slope, and 1005 for E:O.

### 2.5. Statistical Analyses

Descriptive statistics are provided as mean and standard deviation (SD) for data with an assumption of normal distribution, and median and interquartile range for data with an assumption of non-normal distribution. Visualization of the predictor distribution was demonstrated with smooth kernel density plots. Hypothesis testing was carried out to demonstrate the temporal variation in validation cohorts as compared to the development cohorts in each diagnostic endpoint. The independent-sample t-test was used for continuous predictors with a normal distribution assumption, whereas Wilcoxon’s rank-sum test was used for continuous predictors with a non-normal distribution assumption. Fisher’s exact test was used across categorical predictors. A *p*-value of less than 0.05 was considered statistically significant.

A non-parametric receiver-operating characteristic curve was plotted to demonstrate discriminative performance, consisting of the AuROC, with interpretation following the definition of Hosmer–Lemeshow [14]. The calibration plot was used to demonstrate the concurrence of E:O, calibration-in-the-large (CITL), and calibration slope (C-slope) [15]. Decision curve analysis (DCA) was illustrated for visualizing clinical net benefit and net intervention reduction across all ranges of predicted probability [16,17]. The predicted probability based on multivariable binary logistic regression was derived from the previously published article [10]. Re-calibration of the model was carried out according to the C-slope and CITL on the model coefficients and intercept, respectively [18]. No class imbalance method was applied to reflect the true prevalence of elevated LDL-C and elevated non-HDL-C in the validation cohort.

Effects of case-mix and differences in the regression coefficient across development and validation cohorts were demonstrated with three alternative validation strategies, consisting of (1) reference value generation, with the adjustment of case-mix based on the assumption of complete transferability of the regression coefficient to the validation cohort [19,20]; (2) model refitting, which can reflect both the case-mix and true shift of regression coefficients in the validation cohort; (3) metabolic age imputation based on age, gender, and BMI to demonstrate the validity of indirect estimation of metabolic age as a predictor in the validation cohort [10].

Fairness was examined as heterogeneity in AuROC, C-slope, and CITL across various participant characteristics. Subgroup AuROC was demonstrated by the covariate-specific receiver-operating characteristic curve conditioned on the covariate [21]. Subgroup C-slope and CITL were obtained from a binomial generalized linear model with an additional effect modification of C-slope and CITL conditioned on the covariate. The covariates considered in the fairness assessment include gender, BMI, age, fat percentage, muscle percentage, waist-to-height ratio, metabolic age, and DBP. Moreover, the 10 occupation categories from the 2008 International Standard Classification of Occupations (ISCO) [22] were used in the fairness assessment as a surrogate marker of participant lifestyle phenotypes comprising socioeconomic status, health literacy, and occupational risk exposure differences. These combinations of factors were potentially associated with different lipid profiles according to the published literature [23,24]. No missing data was found; hence, no data imputation was carried out. Transparent Reporting of a multivariable prediction model for Individual Prognosis or Diagnosis statement 2024 (TRIPOD-AI 2024) was used as the reporting guidance [25]. All of the analyses and visualizations were performed using STATA version 18.0 (College Station, TX, USA). User-written statistical software components were used as follows: <pmcalplot> for visualizing calibration plots [26]; and <dca> for illustrating DCA plots [16].

### 2.6. Ethics Declaration and Informed Consent Processes

This study, along with the informed consent document and procedure, was approved by the Institutional Review Board of the Faculty of Medicine, Chiang Mai University, Chiang Mai, Thailand, study code: COM-2566-0361, approval date: 20 September 2023. Additionally, to enhance transparency and adhere to evolving best practices, we registered our protocol as an observational study on the Thai Clinical Trial Registry (TCTR), study code: TCTR20240419007, registration date: 19 April 2024. This was conducted in accordance with the recent update from the TRIPOD-AI 2024 statement (item 18b, registration), published on 16 April 2024. All procedures involving human participants were initiated after documented informed consent was obtained, with adherence to the ethical principles set by the World Medical Association’s 1964 Declaration of Helsinki.

## 3. Results

**Figure 1:**
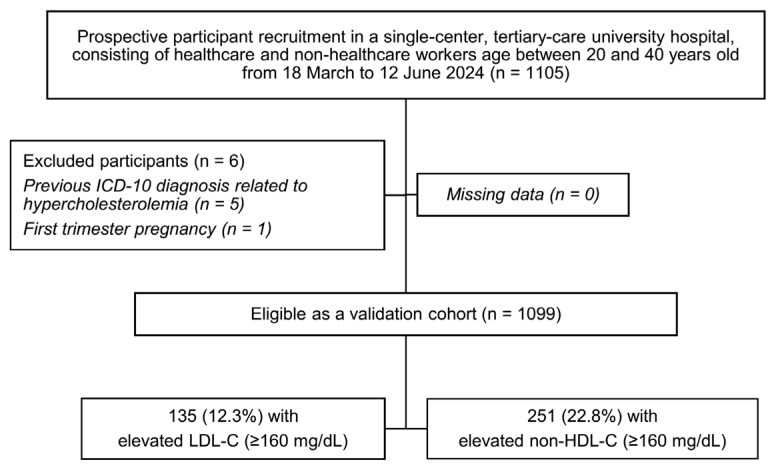
A study flow diagram related to the recruitment of validation cohort; ICD-10, the International Classification of Diseases, Tenth Revision; LDL-C, low-density lipoprotein cholesterol; non-HDL-C, non-high-density lipoprotein cholesterol.

The current study’s inclusion criteria drew a total of 1105 potential participants. The exclusion criteria screened six participants out due to previous ICD-10 diagnoses related to hypercholesterolemia (*n* = 5) and first-trimester pregnancy (*n* = 1). Thereby, the validation cohort consisted of 1099 eligible participants, with 135 (12.3%) elevated LDL-C cases and 251 (22.8%) elevated non-HDL-C cases (Figure 1). A comparison was made with the previous retrospectively recruited development cohort comprising 2222 participants for the LDL-C model with 303 elevated LDL-C cases (13.6%), and 5149 participants for the non-HDL-C model with 1013 elevated non-HDL-C cases (19.7%), as seen in Table 1.

### 3.1. LDL-C Model

**Figure 2:**
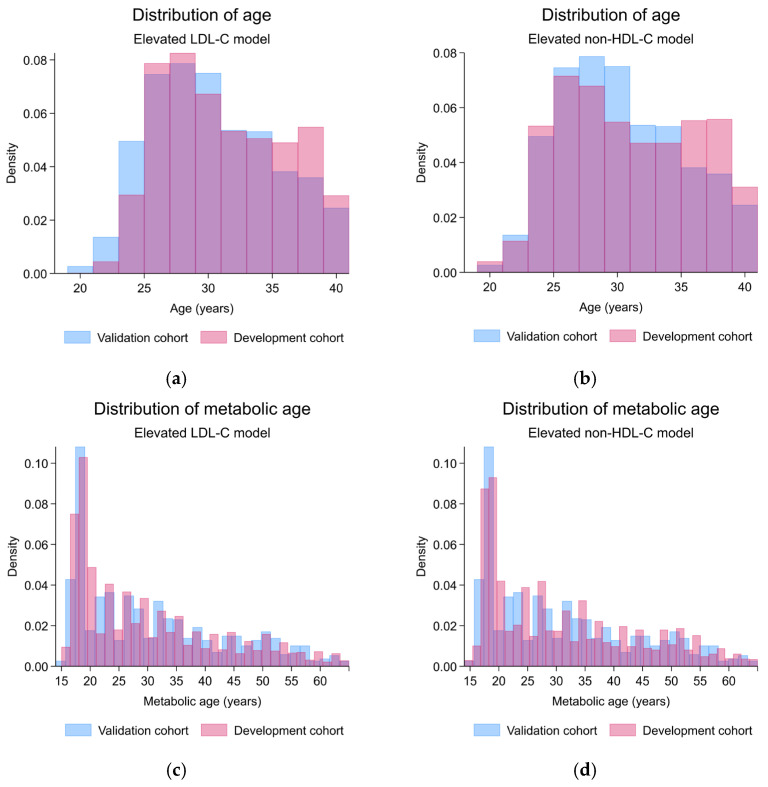

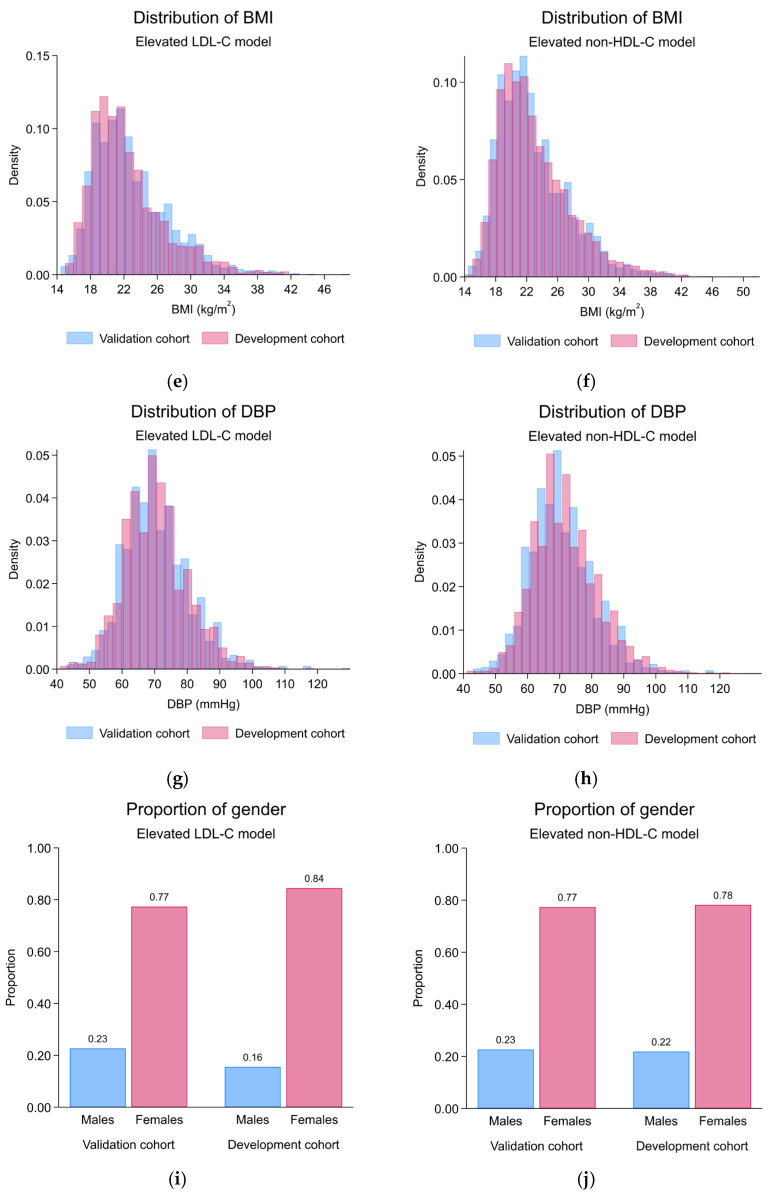

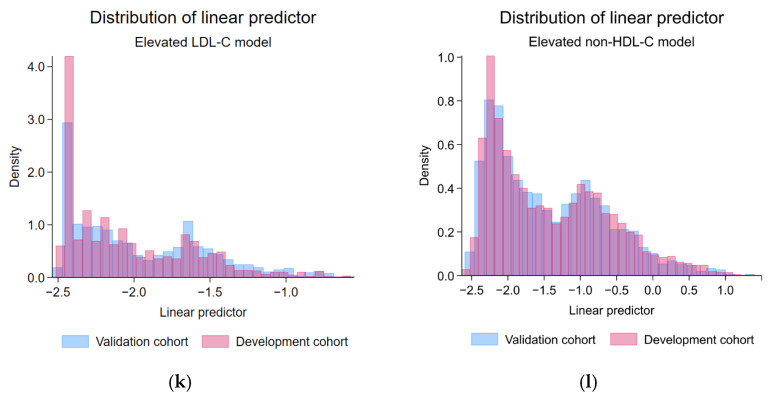
Histogram and bar plots demonstrating the predictor distribution across development and validation cohorts; (**a**) the distribution of age, elevated LDL-C model; (**b**) the distribution of age, elevated non-HDL-C model; (**c**) the distribution of metabolic age, elevated LDL-C model; (**d**) the distribution of metabolic age, elevated non-HDL-C model; (**e**) the distribution of BMI, elevated LDL-C model; (**f**) the distribution of BMI, elevated non-HDL-C model; (**g**) the distribution of DBP, elevated LDL-C model; (**h**) the distribution of DBP, elevated non-HDL-C model; (**i**) the proportion of gender, elevated LDL-C model; (**j**) the proportion of gender, elevated non-HDL-C model; (**k**) the distribution of linear predictor, elevated LDL-C model; (**l**) the distribution of linear predictor, elevated non-HDL-C model; LDL-C, low-density lipoprotein cholesterol; non-HDL-C, non-high-density lipoprotein cholesterol.

The validation cohort demonstrated predictor characteristics with statistically significant differences from the development cohort, which included lower age (mean (SD), years; development: 31.3 (4.7), validation: 30.4 (4.7); *p* < 0.001), a higher proportion of males (count (%); development: 345 (15.5%), validation: 249 (22.7%); *p* < 0.001), higher BMI (mean (SD), kg/m^2^; development: 22.5 (4.5), validation: 22.9 (4.5); *p* = 0.006), higher waist circumference (mean (SD), cm; development: 77.4 (11.2), validation: 79.0 (12.2); *p* < 0.001), higher muscle mass (mean (SD), kg; development: 39.8 (7.9), validation: 41.0 (8.6); *p* < 0.001), higher BMR (mean (SD), kcal/day; development: 1256.0 (233.2), validation: 1290.8 (247.8); *p* < 0.001), higher metabolic age (mean (SD), years; development: 30.1 (12.3), validation: 31.0 (12.6); *p* = 0.04), and higher visceral fat index (mean (SD), units; development: 4.8 (3.4), validation: 5.2 (3.7); *p* = 0.003). However, the same LDL-C level (mean (SD), mg/dL; development: 125.2 (32.1), validation: 123.1 (33.1); *p* = 0.07) and the same LDL-C classification (*p* = 0.30) were found in the validation cohort. The linear predictor from the LDL-C model was significantly higher in the validation cohort (mean (SD); development: −2.04 (0.41), validation: −1.96 (0.45); *p* < 0.001) (Table 1). Visually, the predictor distribution demonstrated minimal practical differences across the development and validation cohorts (Figure 2). Regarding the prediction performances before the model update, discriminative performance was lower in the validation cohort (AuROC 0.59; 95%-CI, 0.56, 0.62) compared to the development cohort (AuROC 0.64; 95%-CI, 0.62, 0.66) (Table 2 and Supplementary File S1, Figure S1A). Miscalibration with the overestimation of risk was found (C-slope 0.64; 95%-CI, 0.39, 0.88 and CITL −0.14; 95%-CI, −0.27, −0.02) (Table 2 and Supplementary File S1, Figure S1C). The model was henceforth updated to the equation as follows:

(1) $$\begin{array}{ll} \mathrm{Predicted} & \;\mathrm{probability}\;\mathrm{of}\;\mathrm{having}\;\mathrm{elevated}\;\mathrm{LDL}-C\\ & \;\;\;\;=\frac{e^{0.5542*(\mathrm{male})+0.0155*(\mathrm{metabolic}\;\mathrm{age}\;(\mathrm{years}))-2.6091}}{1+e^{0.5542*(\mathrm{male})+0.0155*(\mathrm{metabolic}\;\mathrm{age}\;(\mathrm{years}))-2.6091}} \end{array}$$

**Figure 3:**
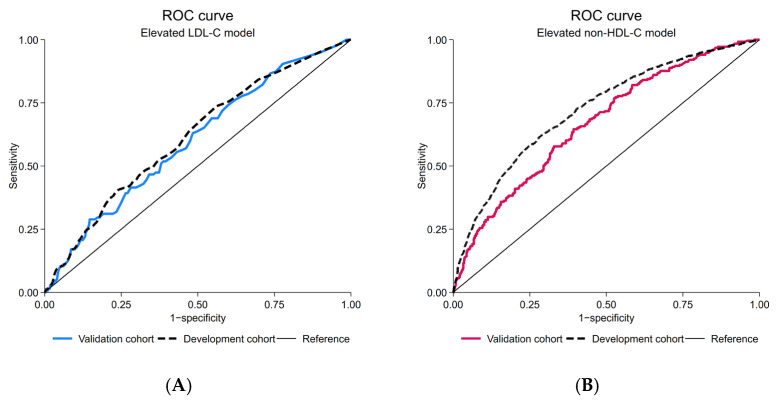

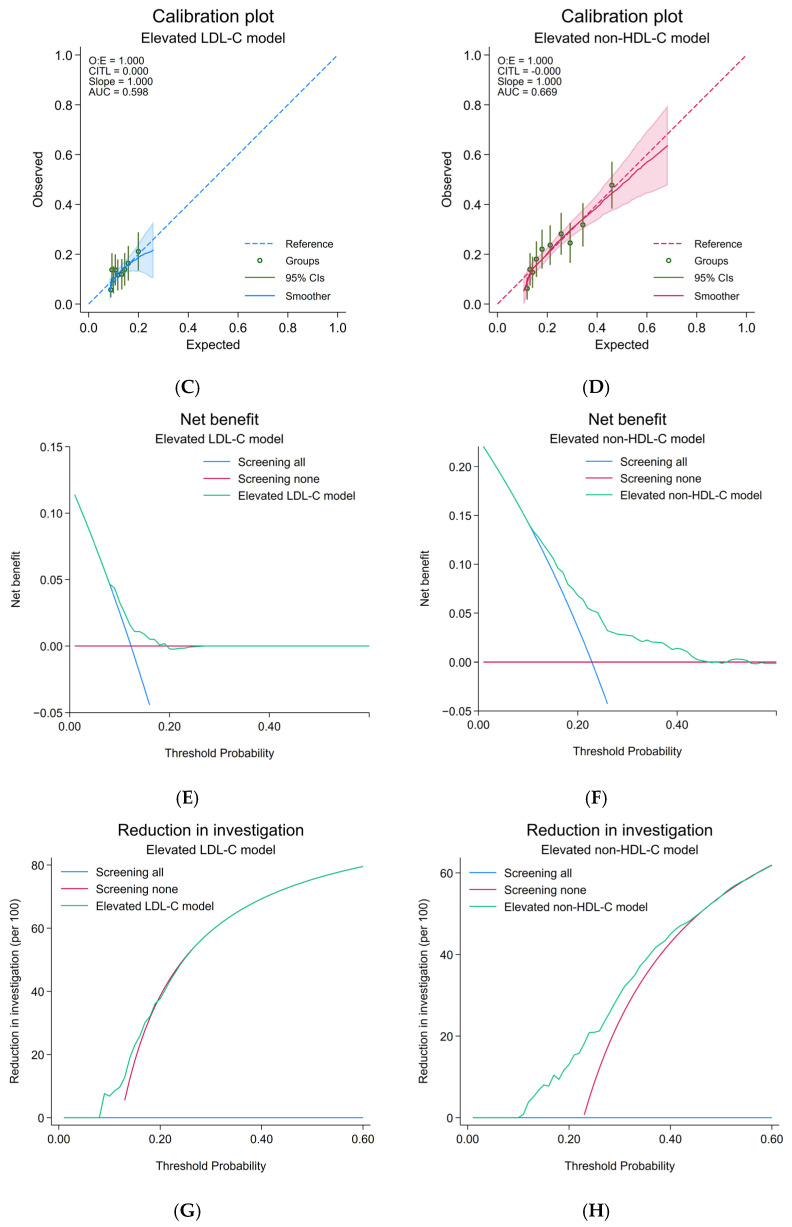
Prediction performances of the updated elevated LDL-C and updated elevated non-HDL-C model; (**A**) receiver-operating characteristic curve, elevated LDL-C model; (**B**) receiver-operating characteristic curve, elevated non-HDL-C model; (**C**) calibration plot, elevated LDL-C model; (**D**) calibration plot, elevated non-HDL-C model; (**E**) decision curve analysis of net benefit, elevated LDL-C model; (**F**) decision curve analysis of net benefit, elevated non-HDL-C model; (**G**) decision curve analysis regarding reduction in number of investigation per 100, elevated LDL-C model; (**H**) decision curve analysis regarding reduction in number of investigation per 100, elevated non-HDL-C model; CIs, confidence intervals; LDL-C, low-density lipoprotein cholesterol; non-HDL-C, non-high-density lipoprotein cholesterol; ROC, receiver-operating characteristic curve.

After the model update, discriminative performance was observed as poor (AuROC 0.59; 95%-CI, 0.56, 0.62) (Figure 3A). The alignment of calibration slope and intercept was achieved (C-slope 0.94; 95%-CI, 0.58, 1.30 and CITL 0.01; 95%-CI, −0.11, 0.13) (Figure 3C). Net positive utility was found in ranges of 10–20% predicted probability (Figure 3E and Supplementary File S1, Figure S1E). Net reduction in investigation was negligible (Figure 3G and Supplementary File S1, Figure S1G).

Exploration into heterogeneity demonstrated that the LDL-C model had worse discrimination and overestimated the probability towards individuals who were classified in ISCO code 3 and 4 subgroups, had higher BMI, higher age, higher fat percentage, lower muscle percentage, higher waist-to-height ratio, higher metabolic age, and lower DBP (Supplementary File S1, Table S1).

### 3.2. Non-HDL-C Model

The validation cohort demonstrated predictor characteristics with statistically significant differences from the development cohort, which included lower age (mean (SD), years; development: 31.0 (5.2), validation: 30.4 (4.7); *p* < 0.001), lower systolic blood pressure (mean (SD), mmHg; development: 116.8 (13.3), validation: 115.5 (13.2); *p* = 0.003), and lower diastolic blood pressure (mean (SD), mmHg; development: 71.9 (10.5), validation: 70.4 (10.1); *p* < 0.001). Moreover, higher serum non-HDL-C levels (mean (SD), mg/dL; development: 131.7 (35.6), validation: 136.5 (36.5); *p* < 0.001) and a higher proportion of the elevated non-HDL-C classification (*p* = 0.018) was found in the validation cohort. The linear predictor from the non-HDL-C model demonstrated no statistically significant differences (mean (SD); development: −1.41 (0.81), validation: −1.43 (0.79); *p* = 0.35) (Table 1). Visually, the predictor distribution demonstrated minimal practical differences across the development and validation cohorts (Figure 2). Regarding the prediction performance metrics, discriminative performance was lower in the validation cohort (AuROC 0.67; 95%-CI, 0.64, 0.69) compared to the development cohort (AuROC 0.72; 95%-CI, 0.70, 0.74) (Table 2 and Supplementary File S1, Figure S1B). Also, the miscalibration with significant overestimation of risk was found particularly in the higher-end of observed probability ranges (C-slope 0.71; 95%-CI, 0.59, 0.83), whereas no significant miscalibration was found in the lower-end of the observed probability ranges (CITL −0.07; 95%-CI, −0.17, 0.03) (Table 2 and Supplementary File S1, Figure S1D).

(2) $$\begin{array}{ll} \mathrm{Predicted} & \;\mathrm{probability}\;\mathrm{of}\;\mathrm{having}\;\mathrm{elevated}\;\mathrm{non}-\mathrm{HDL}-C\\ & \;\;\;=\frac{e^{0.9008*(\mathrm{male})+0.0294*(\mathrm{metabolic}\;\mathrm{age}\;(\mathrm{years}))+0.0100*(\mathrm{DBP}\;(\mathrm{mmHg}))-3.1255}}{1+e^{0.9008*(\mathrm{male})+0.0294*(\mathrm{metabolic}\;\mathrm{age}\;(\mathrm{years}))+0.0100*(\mathrm{DBP}\;(\mathrm{mmHg}))-3.1255}} \end{array}$$

After the model update, discriminative performance was preserved as fair, in line with the level seen originally in the development cohort (AuROC 0.67; 95%-CI, 0.64, 0.69) (Figure 3B). The alignment of calibration slope and intercept was demonstrated (C-slope 0.97; 95%-CI, 0.81, 1.13 and CITL −0.03; 95%-CI, −0.13, 0.07) (Figure 3D). Net positive utility was found in ranges of 10–50% predicted probability (Figure 3F and Supplementary File S1, Figure S1F). Net reduction in investigation was approximately 20% at the prevalence threshold probability (Figure 3H and Supplementary File S1, Figure S1H). A test for the equality of AuROC demonstrated a statistically significant difference between the LDL-C and non-HDL-C model (*p* = 0.026).

**Table 2:** Prediction performances of elevated LDL-C and non-HDL-C models in the validation cohort.

Validation Strategy	LDL-C Model					
	Before Update			After Recalibration of C-Slope and CITL		
	AuROC (95%CI)	C-Slope (95%CI)	CITL (95%CI)	AuROC (95%CI)	C-Slope (95%CI)	CITL (95%CI)
Temporal Validation in the Validation Cohort	0.59 (0.56, 0.62)	0.64 (0.39, 0.88)	−0.14 (−0.27, −0.02)	0.59 (0.56, 0.62)	0.94 (0.58, 1.30)	0.01 (−0.11, 0.13)
Reference value (case-mix adjustment) ^a^	0.63 (0.58, 0.68)	1.01 (0.64, 1.37)	0.00 (−0.18, 0.17)	0.59 (0.54, 0.64)	1.00 (0.41, 1.55)	−0.01 (−0.19, 0.18)
Refitted model in the validation cohort (case-mix adjustment and coefficient re-estimation) ^b^				0.60 (0.55, 0.65)	1.01 (0.45, 1.56)	0.00 (−0.18, 0.18)
Metabolic age from the multivariable linear regression estimation	0.60 (0.55, 0.65)	0.75 (0.35, 1.15)	−0.06 (−0.24, 0.12)	0.60 (0.55, 0.65)	1.11 (0.52, 1.69)	0.01 (−0.17, 0.19)
**Validation Strategy**	**Non**-**HDL**-**C Model**					
	**Before Update**			**After Recalibration of C**-**Slope and CITL**		
	**AuROC** (**95**%**CI**)	**C**-**Slope** (**95**%**CI**)	**CITL** (**95**%**CI**)	**AuROC** (**95**%**CI**)	**C**-**Slope** (**95**%**CI**)	**CITL** (**95**%**CI**)
Temporal Validation in the Validation Cohort	0.67 (0.64, 0.69)	0.71 (0.59, 0.83)	−0.07 (−0.17, 0.03)	0.67 (0.64, 0.69)	0.97 (0.81, 1.13)	−0.03 (−0.13, 0.07)
Reference value (case-mix adjustment) ^a^	0.72 (0.67, 0.75)	1.01 (0.81, 1.22)	0.00 (−0.16, 0.16)	0.66 (0.62, 0.70)	1.00 (0.77, 1.24)	0.00 (−0.15, 0.15)
Refitted model in the validation cohort (case-mix adjustment and coefficient re-estimation) ^b^				0.67 (0.63, 0.71)	0.98 (0.74, 1.22)	0.01 (−0.14, 0.15)
Metabolic age from the multivariable linear regression estimation	0.66 (0.62, 0.70)	0.74 (0.55, 0.92)	0.10 (−0.05, 0.25)	0.66 (0.62, 0.70)	1.01 (0.75, 1.26)	0.03 (−0.12, 0.17)

Notes: ^a^ 1000 replications of 1099 participants in the validation cohort; case-mix-corrected AuROC, C-slope, and CITL with bootstrapped 95% CIs. ^b^ Blank cells due to non-applicable summary data. Abbreviations: AuROC, area under the receiver-operating characteristic curve; CI, confidence interval; CITL, calibration-in-the-large; C-slope, calibration slope; LDL-C, low-density lipoprotein cholesterol; non-HDL-C, non-high-density lipoprotein cholesterol.

Exploration into heterogeneity demonstrated that the non-HDL-C model had worse discrimination and overestimated the probability towards individuals who were classified in ISCO code 3 and 4 subgroups, had lower BMI, lower age, higher fat percentage, lower muscle percentage, higher waist-to-height ratio, lower metabolic age, and lower DBP (Supplementary File S1, Table S2).

## 4. Discussion

Diagnostic prediction models should only be used in public health and clinical practice when validation in the source population intended for usage has been extensively carried out [27]. The results from our study, thus, added to the information by demonstrating a degree of reproducibility across both recently developed models, predicting the endpoints consisting of elevated LDL-C and elevated non-HDL-C. Though with a slight difference in time-varying demographic and anthropometric predictor characteristics, the prediction performance was observed to be considerably reduced in both models compared to the original literature [10]. According to the results, the LDL-C model has poor prediction performance across all measures, whereas the non-HDL-C model provides fair discriminative performance with mild miscalibration restricted to the higher ranges of predicted probability. Due to the lower estimated AuROC for the LDL-C model compared to the non-HDL-C model from the starting point in the development cohort, we subsequently found small but statistically significant AuROC differences across the two models in the validation cohort. This pointed out a persistent disparity in discriminative performance across the two models. In terms of clinical significance, however, the clinical net benefit demonstrated a considerable difference in investigation reduction, with negligible reduction at the prevalence threshold probability for the LDL-C model compared with 20% reduction for the non-HDL-C model. Finally, the reason for this improved performance was probably from the inclusion of additional information from DBP, which is known to be associated with a higher non-HDL-C [28]. We suspected that blood pressure, specifically DBP, acted as an objectively measured surrogate predictor for regular exercise. In a population consisting of young adults, DBP but not SBP was observed to be higher in individuals with a sedentary lifestyle. Nevertheless, the improvement in prediction was not large due to an observed small magnitude of association between DBP and exercise as an intervention, especially in healthy, young, normotensive individuals [29,30].

We suspected that too low a proportion of participants in both the development and validation cohorts in the higher ranges of predicted probability led to miscalibration based on the locally smoothed calibration line [15]. Moreover, neither the reference value nor the refitting method revealed that the differences in case-mix or regression coefficient can explain the variation in prediction performances across the development and validation cohorts. We, therefore, ultimately suspect that the major causes are the intrinsically limited ability of anthropometric predictors to predict elevated LDL-C and non-HDL-C endpoints [19,20]. These results aligned with the previous large Chinese studies reporting the discrimination of univariable anthropometric measurements and abnormality in lipid profiles, in which poor-to-fair point estimates of AuROC were found, ranging from 0.586 to 0.659 for LDL-C, 0.575 to 0.629 for total cholesterol, and 0.646 for dyslipidemia [31,32]. In addition, results from the analysis of the United States 2017–2018 NHANES also found poor-to-fair non-HDL-C discrimination performance, with point AuROC estimated as ranging from 0.59 to 0.71 for both the raw values and quartiles of waist circumference and BMI [33]. In summary, the potential unmeasured variables—with high challenges in gathering objective measurements such as genetics and lifestyle factors—may have a considerable influence on the prediction of lipid profiles beyond that of the anthropometric measurements alone. Polygenic risk score or reliable measurements of dietary intake may help as a novel predictor in the future [34,35].

Nevertheless, more evidence in support of the real-world application of the non-HDL-C model came from the net benefit derived from DCA [17]. We suggest that public health and clinical practice can potentially benefit from using the non-HDL-C model for screening instead of the conventional strategy of either screen all or screen none by setting the pre-specified threshold probability within ranges of model positive clinical net utility for directing decisions for at-risk individuals to confirm their status with the standard serum lipid investigation. For example, the threshold probability at prevalence (22.8%) may potentially translate into a reduction in non-HDL-C investigation by 20%. The impact could translate into one in five phlebotomy and laboratory tests saved compared to the default strategy consisting of entire population screening. The result, therefore, can assist healthcare optimization in situations where there are limited resources. Nevertheless, the threshold could be set to a lower or higher probability than prevalence but within the ranges of clinical net benefit shown for a flexible application across a wide range of policy-making [16,17].

The main limitation of this study is the small number of cases used for testing the calibration slope in the LDL-C model due to the relatively low prevalence of elevated LDL-C cases in the young adult population recruited in this study. Moreover, the high healthcare accessibility of our source population from working in hospital settings may have affected the pool of eligible participants by filtering out a larger number of previously ICD-10-diagnosed cases than would happen in a normal young adult population when applying the exclusion criteria. Therefore, we suggested that, in order to generalize the prediction performances with confidence, the implemented population should have a relatively similar distribution of basic predictors, such as age and gender, compared to this study. Such relatable population characteristics were observed across many studies across the country [36,37], where the tertiary care workforce was a female majority with ages primarily in the third to fourth decades. However, the heterogeneity related to performance was observed in subgroups and sub-phenotypes of participants in both models, which may have resulted in an overestimation of probability and a higher chance of false-positive predictions. Notably, in the non-HDL-C model, the profile of heterogeneity was suggestively aligned with the sarcopenic obesity phenotype, where BMI may not be as high as the traditional case of obesity—resulting from the combination of lower muscle mass and higher fat mass—but indirectly evident in a higher waist-to-height ratio [38]. It has been known that pre-sarcopenia in urban residents has an early onset and is present in one-fourth of the younger Thai population based on a study conducted using dual-energy X-ray absorptiometry (DXA) [39]. Lastly, the metabolic age, which was calculated from age, gender, and BMI, may not alone be able to capture the full picture of this phenotype. Therefore, an external validation aiming at a model update with new predictors or an alternative strategy of a stratified prediction model might need to be tested in these certain phenotypes deviated from the traditional cases of obesity to provide valid prediction performance [40].

Additionally, metabolic age may not be available in every practice setting. In this case, the method for estimating unavailable metabolic age in the validation cohort was derived from multivariable linear regression using age, gender, and BMI (Supplementary File S1, Figure S2) according to the equation listed in our previous article—which is demonstrated in the current article for replicability purposes and also added on as an intrinsic, auxiliary function in the web-based calculator: https://wuttipatk.shinyapps.io/ShinyR2/ (accessed on 26 October 2025) [10]. The estimation in the validation cohort demonstrated adequate calibration for estimation in the ranges of possible metabolic age (N = 1099, R^2^ = 0.87, CITL = 0.57, C-slope 1.05). Furthermore, the current web-based calculator equations predicting elevated LDL-C and non-HDL-C were the updated models related to this research, with temporal validation applied. Intended users of the calculator may include healthcare practitioners and the general population who have access to the internet and wish to precisely calculate the risk for individualized prediction. The results could assist in making an early decision regarding further confirmation of one’s lipid profile in high-risk cases.

(3) $$\begin{array}{ll} \mathrm{Metabolic}\;\mathrm{age} & \;\mathrm{estimation}\;\mathrm{in}\;\mathrm{years}\\ & \;=0.541\times \left(\mathrm{Age}\;\mathrm{in}\;\mathrm{years}\right)+2.394\times \left(\mathrm{Body}\;\mathrm{mass}\;\mathrm{index}\;\mathrm{in}\;\mathrm{kg}/m^{2}\right)\\ & \;-7.326\times \left(\mathrm{Male}\;\mathrm{gender}\right)-39.156 \end{array}$$

The prediction performances were demonstrated to be robust to metabolic age imputation by the aforementioned method. The imputation using universally available predictors will be beneficial for remote areas and primary care adaptation of the current model, where limited access to a body composition analyzer is highly probable. This can lead to the scaling up of the model implementation and maximizing usage of routinely collected retrospective data by village health volunteers and health officers in rural primary care settings for the triaging of elevated non-HDL-C cases across the country. Moreover, local clinical practice guidelines may also incorporate the model as a part of decision-making processes, tailoring the threshold probability according to the available medical resources. For example, a higher model threshold probability than the population prevalence that falls within the ranges of clinical net benefit may be used in areas with limited resources for confirmatory laboratory non-HDL-C investigation. An additional strength of our study is the pre-registered protocol, which provides the framework for the prospective recruitment of participants, which augments our quality of data by both standardizing the measurement of predictors and ensuring a minimal amount of missing data. Thereby, the threat to the internal validity of this study, including measurement error and selection bias from excluding incomplete cases, is minimized [41].

From the performance measures across both models, we suggest that future research should be focused solely on the geographical and domain external validation of the non-HDL-C model, in which the data collection should focus on a broader domain, especially young adults in approximately the same age bracket but employed in other occupational activities separate from the currently predominant group of service sectors. Alternatively, the research could also assess and update models in ISCO subgroups with poor performances, particularly the ISCO codes 3 and 4, or participants presenting with a normal BMI with low muscle and high fat mass related to sarcopenic obesity. The study should have an adequate number of samples to avoid inferior prediction performance, particularly with discrimination and calibration, when compared to the current study’s overall prediction performance. Additionally, an effectiveness study should be launched to investigate how the non-HDL-C model acts as a directive strategy for public health and clinical practice in screening, compared to the current standard of care in capturing high-risk individuals. The ideal type of effectiveness study should be a cluster-randomized controlled concurrent trial, or, less ideally, the before–after study comparing a group of people receiving screening assistance by the non-HDL-C model versus the usual care process, to compare performance measures and cost-effectiveness properties of the model [42].

## 5. Conclusions

Our research highlights how diagnostic prediction models can both help increase accessibility and acknowledgement of elevated non-HDL-C in young adults. The major goals of temporal validation demonstrated that the LDL-C model has negligible benefit in prediction. However, the non-HDL-C model with predictors consisting of gender, metabolic age, and DBP may assist public health and clinical practice in case detection, though with limitations in populations with a high proportion of phenotypes presenting with a normal BMI with low muscle and high fat mass related to sarcopenic obesity. Nonetheless, the non-HDL-C model may possibly be utilized as a part of a non-invasive triage strategy before official laboratory confirmation of the lipid profile and, consequently, could facilitate the initiation of proper ASCVD primary prevention in young adults at risk within the source population.

## Figures and Tables

**Table 1:** Participant characteristics of development and validation cohort across two models, elevated LDL-C and non-HDL-C Model.

Predictors	Validation Cohort	LDL-C Model Development Cohort	*p*-Value	Non-HDL-C Development Cohort	*p*-Value
	N = 1099	N = 2222		N = 5149	
Age (years) ^a^	30.4 (4.7)	31.3 (4.7)	<0.001	31.0 (5.2)	<0.001
Gender ^b^					
Female	850 (77.3%)	1877 (84.5%)	<0.001	4024 (78.2%)	0.57
Male	249 (22.7%)	345 (15.5%)		1125 (21.8%)	
Body mass index (kg/m^2^) ^a^	22.9 (4.5)	22.5 (4.5)	0.006	23.0 (4.8)	0.44
Waist circumference (cm) ^a^	79.0 (12.2)	77.4 (11.2)	<0.001	79.4 (12.1)	0.32
Fat mass (kg) ^a^	16.9 (8.3)	16.4 (8.3)	0.12	17.0 (8.9)	0.53
Muscle mass (kg) ^a^	41.0 (8.6)	39.8 (7.9)	<0.001	41.2 (8.9)	0.47
Basal metabolic rate (kcal/day) ^a^	1290.8 (247.8)	1256.0 (233.2)	<0.001	1295.9 (259.6)	0.56
Metabolic age (years) ^a^	31.0 (12.6)	30.1 (12.3)	0.041	31.1 (12.9)	0.83
Visceral fat index (point) ^a^	5.2 (3.7)	4.8 (3.4)	0.003	5.3 (3.8)	0.25
Systolic blood pressure (mmHg) ^a^	115.5 (13.2)	115.3 (12.6)	0.64	116.8 (13.3)	0.003
Diastolic blood pressure (mmHg) ^a^	70.4 (10.1)	70.5 (9.9)	0.78	71.9 (10.5)	<0.001
**Diagnostic Endpoints**	**Validation Cohort**	**LDL** **-C Model** **Development Cohort**	***p*** **-Value**	**Non** **-HDL** **-C** **Development Cohort**	***p*** **-Value**
	**N = 1099**	**N = 2222**		**N = 5149**	
Serum LDL-C (mg/dL) ^a,c^	123.1 (33.1)	125.2 (32.1)	0.072		
LDL-C classification ^b,c^					
Normal (<160 mg/dL)	964 (87.7%)	1919 (86.4%)	0.3		
Elevated (≥160 mg/dL)	135 (12.3%)	303 (13.6%)			
Linear predictor of LDL-C ^a,c^	−1.96 (0.45)	−2.04 (0.41)	<0.001		
Serum non-HDL-C (mg/dL) ^a,c^	136.5 (36.5)			131.7 (35.6)	<0.001
Non-HDL-C classification ^b,c^					
Normal (<160 mg/dL)	848 (77.2%)			4136 (80.3%)	0.018
Elevated (≥160 mg/dL)	251 (22.8%)			1013 (19.7%)	
Linear predictor of non-HDL-C ^a,c^	−1.43 (0.79)			−1.41 (0.81)	0.35

Notes: ^a^ Mean (SD), *p*-value derived from two independent-samples t-tests, comparing between validation and development cohort. ^b^ Count (percentage), *p*-value derived from Fisher’s exact test, comparing between validation and development cohorts. ^c^ Blank cells due to non-applicable summary data. Abbreviations: kcal, kilocalories per day; kg/m^2^, kilogram per square meters; LDL-C, low-density lipoprotein cholesterol; mg/dL, milligram per deciliters; mmHg, millimeters of mercury; non-HDL-C, non-high-density lipoprotein cholesterol.

## Data Availability

De-identified individual participant data and analytic codes are available upon reasonable request to the corresponding author.

## Supplementary Materials

The following supporting information can be downloaded at https://www.mdpi.com/article/10.3390/jcm14217617/s1, Figure S1. Prediction performances of the pre-updated elevated LDL-C and updated elevated non-HDL-C model: (a) receiver-operating characteristic curve, elevated LDL-C model; (b) receiver-operating characteristic curve, elevated non-HDL-C model; (c) calibration plot, elevated LDL-C model; (d) calibration plot, elevated non-HDL-C model; (e) decision curve analysis of net benefit, elevated LDL-C model; (f) decision curve analysis of net benefit, elevated non-HDL-C model; (g) decision curve analysis regarding reduction in number of investigation per 100, elevated LDL-C model; (h) decision curve analysis regarding reduction in number of investigation per 100, elevated non-HDL-C model; CIs, confidence intervals; CITL, calibration-in-the-large; LDL-C, low-density lipoprotein cholesterol; non-HDL-C, non-high-density lipoprotein cholesterol; O:E, observed to expected ratio; ROC, receiver-operating characteristic curve; Figure S2. Calibration plots of observed metabolic age and imputed metabolic age in the validation cohort; CITL, calibration-in-the-large; Table S1. Subgroup analyses based on participant characteristics, elevated LDL-C model; BMI, body mass index; cm, centimeters; CI, confidence interval; DBP, diastolic blood pressure; ISCO, International Standard Classification of Occupations; kcal, kilocalories per day; kg/m^2^, kilogram per square meters; LDL-C, low-density lipoprotein cholesterol; mg/dL, milligram per deciliters; mmHg, millimeters of mercury; Table S2. Subgroup analyses based on participant characteristics, elevated LDL-C model; BMI, body mass index; cm, centimeters; CI, confidence interval; DBP, diastolic blood pressure; ISCO, International Standard Classification of Occupations; kcal, kilocalories per day; kg/m^2^, kilogram per square meters; mg/dL, milligram per deciliters; mmHg, millimeters of mercury; non-HDL-C, non-high-density lipoprotein cholesterol.

## Abbreviations

The following abbreviations are used in this manuscript:

aRR	adjusted risk ratio
ASCVD	atherosclerotic cardiovascular disease
AuROC	area under the receiver-operating characteristic curve
BIA	bioelectrical impedance analysis
BMI	body mass index
CITL	calibration-in-the-large
CI	confidence interval
C-slope	calibration slope
cm	centimeters
DBP	diastolic blood pressure
DCA	decision curve analysis
E:O	observed to expected ratio
ICD-10	International Classification of Diseases, Tenth Revision
ISCO	International Standard Classification of Occupations
Kcal	kilocalories per day
kg/m^2^	kilogram per square meter
LDL-C	low-density lipoprotein cholesterol
mg/dL	milligram per deciliter
mmHg	millimeters of mercury
Non-HDL-C	non-high-density lipoprotein cholesterol
PCSK9	proprotein convertase subtilisin/kexin type 9
ROC	receiver-operating characteristic curve
SBP	systolic blood pressure
TRIPOD-AI	Transparent Reporting of a multivariable prediction model for Individual Prognosis or Diagnosis using artificial intelligence
