# Supplementary material for: A Temporal Validation Study of Diagnostic Prediction Models for the Screening of Elevated Low-Density and Non-High-Density Lipoprotein Cholesterol
Source: J Clin Med. 2025 Oct 27;14(21):7617. doi: 10.3390/jcm14217617 (PMC12609855; doi:10.3390/jcm14217617)
Supplement: Supplementary file 1 [file jcm-14-07617-s001.zip › jcm-3932034-supplementary.pdf]

Additional file 1

Figure S1. Prediction performances of the pre-updated elevated LDL-C and updated elevated non-HDL-C model; **(a)** Receiver-operating characteristic curve, elevated LDL-C model; **(b)** Receiver-operating characteristic curve, elevated non-HDL-C model; **(c)** Calibration plot, elevated LDL-C model; **(d)** Calibration plot, elevated non-HDL-C model; **(e)** Decision curve analysis of net benefit, elevated LDL-C model; **(f)** Decision curve analysis of net benefit, elevated non-HDL-C model; **(g)** Decision curve analysis regarding reduction in number of investigation per 100, elevated LDL-C model; **(h)** Decision curve analysis regarding reduction in number of investigation per 100, elevated non-HDL-C model; CIs, confidence intervals; CITL, calibration-in-the-large; LDL-C, low-density lipoprotein cholesterol; non-HDL-C, non-high-density lipoprotein cholesterol; O:E, observed to expected ratio; ROC, receiver-operating characteristic curve;

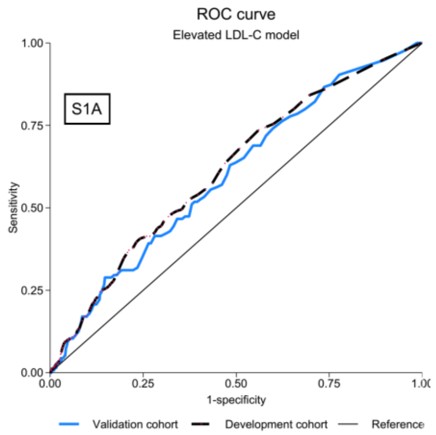

(a)

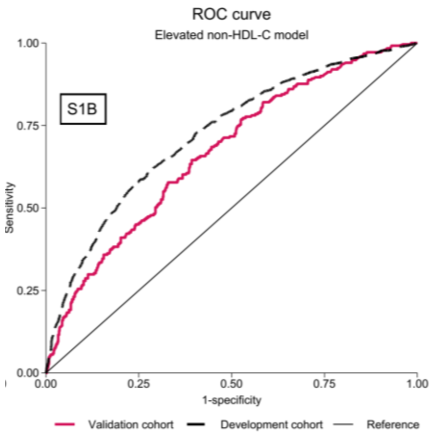

(b)

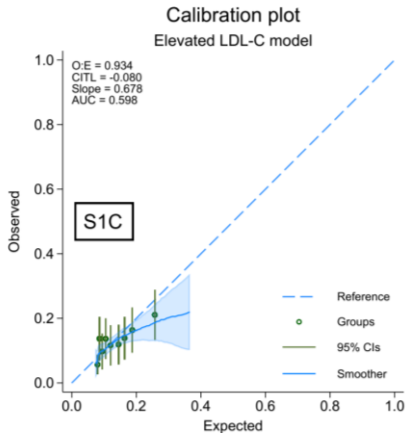

(c)

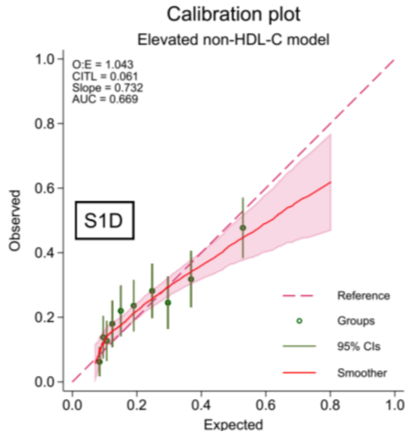

(d)

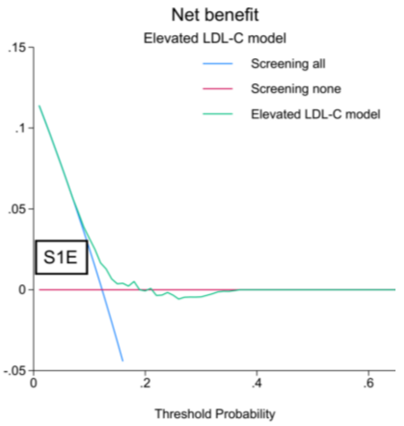

(e)

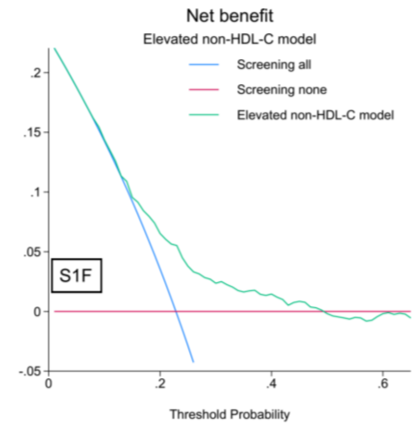

(f)

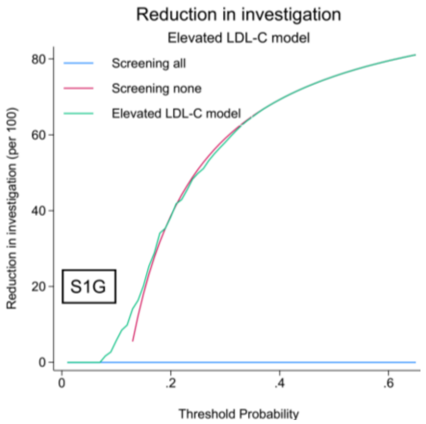

(g)

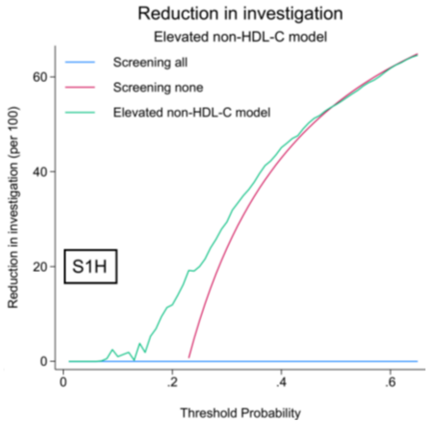

(h)

Figure S2. Calibration plots of observed metabolic age and imputed metabolic age in the validation cohort; CITL, calibration-in-the-large;

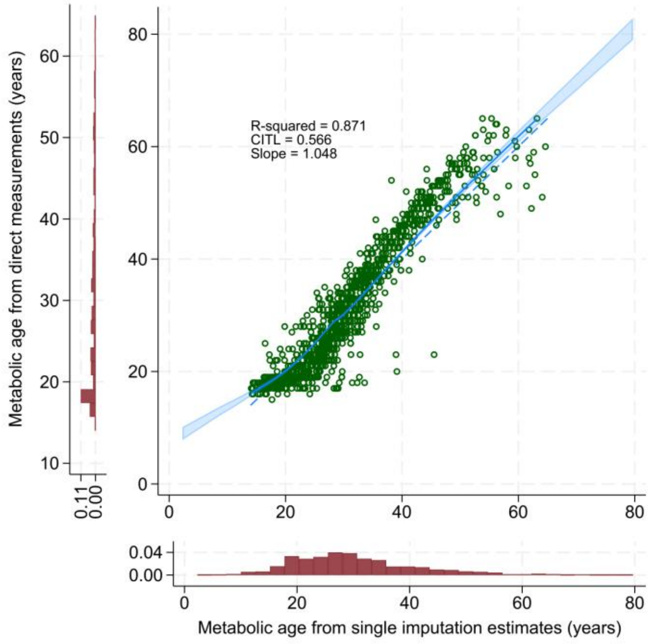

Table S1. Subgroup analyses based on participant characteristics, elevated LDL-C model; BMI, body mass index; cm, centimeters; CI, confidence interval; DBP, diastolic blood pressure; ISCO, International Standard Classification of Occupations (ISCO); kcal, kilocalories per day; kg/m², kilogram per square meters; LDL-C, low-density lipoprotein cholesterol; mg/dL, milligram per deciliters; mmHg, millimeters of mercury;

	Elevated LDL-C model					
	Before update			After recalibration of c-slope and CITL		
**Subgroup analyses**	**AuROC (95%CI)**	**C-slope (95%CI)**	**CITL (95%CI)**	**AuROC (95%CI)**	**C-slope (95%CI)**	**CITL (95%CI)**
Gender						
Female subgroup	0.57 (0.52, 0.63)	0.73 (0.02, 1.44)	-0.01 (-0.23, 0.21)	0.56 (0.50, 0.62)	1.08 (0.04, 2.12)	-0.01 (-0.23, 0.21)
Male subgroup	0.51 (0.40, 0.62)	0.29 (-0.85, 1.43)	-0.23 (-0.55, 0.10)	0.51 (0.41, 0.62)	0.42 (-1.26, 2.10)	0.03 (-0.30, 0.35)
**ISCO code**						
ISCO code 2 subgroup	0.63 (0.55, 0.71)	1.23 (0.62, 1.83)	-0.01 (-0.26, 0.25)	0.63 (0.55, 0.71)	1.81 (0.92, 2.70)	0.02 (-0.23, 0.28)
ISCO code 3 subgroup	0.48 (0.38, 0.59)	-0.09 (-0.93, 0.74)	-0.13 (-0.51, 0.26)	0.48 (0.38, 0.59)	-0.14 (-1.37, 1.10)	-0.04 (-0.42, 0.34)
ISCO code 4 subgroup	0.55 (0.39, 0.71)	0.22 (-0.97, 1.41)	0.07 (-0.45, 0.59)	0.55 (0.39, 0.71)	0.33 (-1.43, 2.08)	0.17 (-0.35, 0.69)
ISCO code 9 subgroup	0.58 (0.41, 0.74)	0.88 (-0.56, 2.32)	-0.38 (-0.95, 0.19)	0.58 (0.41, 0.74)	1.29 (-0.83, 3.42)	-0.16 (-0.73, 0.40)
Other ISCO subgroup (1,5,6,7)	0.74 (0.47, 1.01)	1.63 (-0.37, 3.64)	-0.26 (-1.13, 0.61)	0.74 (0.47, 1.01)	2.41 (-0.55, 5.36)	-0.10 (-0.96, 0.76)
**BMI**						
BMI, 15 kg/m²	0.59 (0.47, 0.71)	1.15 (0.24, 2.06)	0.10 (-0.29, 0.48)	0.59 (0.47, 0.71)	1.69 (0.35, 3.04)	-0.03 (-0.41, 0.34)
BMI, 20 kg/m²	0.56 (0.49, 0.63)	0.84 (0.21, 1.47)	0.00 (-0.24, 0.23)	0.56 (0.49, 0.63)	1.23 (0.30, 2.17)	-0.01 (-0.24, 0.22)
BMI, 25 kg/m²	0.53 (0.47, 0.59)	0.53 (-0.05, 1.10)	-0.10 (-0.29, 0.09)	0.54 (0.47, 0.60)	0.78 (-0.07, 1.62)	0.00 (-0.18, 0.19)
BMI, 30 kg/m²	0.50 (0.39, 0.62)	0.22 (-0.57, 1.01)	-0.20 (-0.50, 0.10)	0.51 (0.39, 0.63)	0.32 (-0.84, 1.48)	0.02 (-0.27, 0.32)
BMI, 35 kg/m²	0.47 (0.29, 0.65)	-0.09 (-1.22, 1.04)	-0.30 (-0.77, 0.17)	0.49 (0.31, 0.66)	-0.13 (-1.80, 1.53)	0.04 (-0.42, 0.51)
BMI, 40 kg/m²	0.44 (0.21, 0.68)	-0.40 (-1.91, 1.11)	-0.40 (-1.05, 0.25)	0.46 (0.22, 0.70)	-0.59 (-2.82, 1.64)	0.06 (-0.58, 0.70)
**Age**						
Age, 20 years old	0.62 (0.49, 0.74)	0.74 (-0.29, 1.76)	-0.08 (-0.54, 0.38)	0.61 (0.49, 0.73)	1.09 (-0.42, 2.60)	-0.11 (-0.57, 0.34)
Age, 25 years old	0.60 (0.52, 0.67)	0.69 (0.03, 1.36)	-0.08 (-0.37, 0.21)	0.60 (0.52, 0.67)	1.02 (0.04, 2.00)	-0.06 (-0.35, 0.23)
Age, 30 years old	0.58 (0.53, 0.63)	0.65 (0.23, 1.07)	-0.08 (-0.27, 0.11)	0.58 (0.53, 0.63)	0.96 (0.34, 1.58)	-0.01 (-0.19, 0.18)
Age, 35 years old	0.56 (0.48, 0.64)	0.61 (0.10, 1.11)	-0.08 (-0.32, 0.16)	0.56 (0.49, 0.64)	0.90 (0.15, 1.64)	0.04 (-0.20, 0.28)
Age, 40 years old	0.54 (0.42, 0.67)	0.56 (-0.26, 1.38)	-0.08 (-0.48, 0.31)	0.55 (0.42, 0.67)	0.83 (-0.38, 2.04)	0.10 (-0.29, 0.49)
**Fat percentage**						
Fat percentage, 10%	0.59 (0.48, 0.70)	1.83 (0.58, 3.09)	-0.04 (-0.45, 0.36)	0.59 (0.48, 0.70)	2.70 (0.85, 4.55)	0.00 (-0.41, 0.40)
Fat percentage, 20%	0.59 (0.52, 0.66)	1.10 (0.51, 1.70)	-0.07 (-0.30, 0.17)	0.59 (0.52, 0.66)	1.63 (0.75, 2.50)	0.00 (-0.24, 0.24)

Fat percentage, 30%	0.59 (0.54, 0.64)	0.37 (-0.13, 0.88)	-0.09 (-0.28, 0.10)	0.59 (0.54, 0.64)	0.55 (-0.20, 1.30)	0.00 (-0.19, 0.19)
Fat percentage, 40%	0.59 (0.52, 0.65)	-0.35 (-1.49, 0.78)	-0.11 (-0.42, 0.21)	0.59 (0.53, 0.65)	-0.52 (-2.20, 1.15)	0.00 (-0.32, 0.32)
Fat percentage, 50%	0.58 (0.49, 0.68)	-1.08 (-2.94, 0.77)	-0.13 (-0.63, 0.37)	0.59 (0.49, 0.69)	-1.60 (-4.34, 1.14)	0.01 (-0.50, 0.51)
**Muscle percentage**						
Muscle percentage, 50%	0.59 (0.50, 0.67)	-0.92 (-2.57, 0.72)	-0.12 (-0.56, 0.32)	0.58 (0.50, 0.67)	-1.36 (-3.78, 1.06)	0.00 (-0.44, 0.44)
Muscle percentage, 60%	0.59 (0.53, 0.64)	-0.13 (-1.02, 0.77)	-0.10 (-0.36, 0.16)	0.59 (0.53, 0.64)	-0.19 (-1.51, 1.13)	0.00 (-0.26, 0.26)
Muscle percentage, 70%	0.59 (0.54, 0.65)	0.67 (0.27, 1.07)	-0.08 (-0.26, 0.11)	0.59 (0.54, 0.65)	0.99 (0.40, 1.58)	0.00 (-0.18, 0.18)
Muscle percentage, 80%	0.59 (0.51, 0.68)	1.47 (0.58, 2.35)	-0.06 (-0.36, 0.25)	0.60 (0.51, 0.68)	2.16 (0.86, 3.47)	0.00 (-0.31, 0.31)
Muscle percentage, 90%	0.59 (0.47, 0.72)	2.26 (0.63, 3.89)	-0.03 (-0.53, 0.46)	0.60 (0.47, 0.73)	3.34 (0.94, 5.74)	0.00 (-0.50, 0.50)
**Waist-to-height ratio**						
Waist-to-height ratio, 0.4	0.56 (0.47, 0.65)	0.99 (0.22, 1.76)	0.07 (-0.24, 0.38)	0.55 (0.46, 0.64)	1.46 (0.32, 2.60)	-0.01 (-0.32, 0.30)
Waist-to-height ratio, 0.5	0.54 (0.48, 0.60)	0.65 (0.08, 1.22)	-0.08 (-0.26, 0.11)	0.54 (0.48, 0.60)	0.96 (0.11, 1.80)	0.00 (-0.18, 0.18)
Waist-to-height ratio, 0.6	0.52 (0.40, 0.64)	0.31 (-0.46, 1.09)	-0.22 (-0.53, 0.09)	0.53 (0.42, 0.65)	0.46 (-0.68, 1.60)	0.01 (-0.30, 0.32)
Waist-to-height ratio, 0.7	0.50 (0.30, 0.70)	-0.03 (-1.21, 1.16)	-0.37 (-0.91, 0.16)	0.53 (0.33, 0.72)	-0.04 (-1.79, 1.71)	0.02 (-0.51, 0.55)
Waist-to-height ratio, 0.8	0.48 (0.21, 0.75)	-0.37 (-2.02, 1.29)	-0.52 (-1.30, 0.25)	0.52 (0.25, 0.79)	-0.54 (-2.99, 1.91)	0.03 (-0.74, 0.80)
**Metabolic age**						
Metabolic age, 20 years	0.61 (0.53, 0.69)	1.00 (0.39, 1.62)	0.04 (-0.22, 0.29)	0.61 (0.51, 0.71)	1.48 (0.57, 2.38)	0.02 (-0.23, 0.27)
Metabolic age, 30 years	0.54 (0.49, 0.59)	0.77 (0.28, 1.25)	-0.05 (-0.24, 0.13)	0.54 (0.48, 0.59)	1.13 (0.41, 1.84)	0.00 (-0.18, 0.19)
Metabolic age, 40 years	0.47 (0.39, 0.55)	0.53 (-0.03, 1.09)	-0.14 (-0.35, 0.07)	0.47 (0.38, 0.56)	0.78 (-0.05, 1.60)	-0.01 (-0.22, 0.20)
Metabolic age, 50 years	0.40 (0.27, 0.53)	0.29 (-0.49, 1.07)	-0.23 (-0.53, 0.07)	0.40 (0.25, 0.55)	0.43 (-0.72, 1.58)	-0.03 (-0.33, 0.27)
Metabolic age, 60 years	0.33 (0.15, 0.51)	0.05 (-1.01, 1.12)	-0.31 (-0.73, 0.11)	0.33 (0.12, 0.55)	0.08 (-1.49, 1.65)	-0.05 (-0.46, 0.37)
**DBP**						
DBP, 50 mmHg	0.56 (0.44, 0.67)	0.72 (-0.22, 1.66)	-0.13 (-0.56, 0.29)	0.55 (0.44, 0.66)	1.06 (-0.32, 2.45)	-0.20 (-0.62, 0.22)
DBP, 60 mmHg	0.56 (0.49, 0.63)	0.66 (0.01, 1.30)	-0.11 (-0.39, 0.17)	0.56 (0.49, 0.63)	0.97 (0.01, 1.92)	-0.11 (-0.38, 0.17)
DBP, 70 mmHg	0.57 (0.51, 0.62)	0.59 (0.14, 1.04)	-0.09 (-0.27, 0.10)	0.57 (0.51, 0.62)	0.87 (0.21, 1.53)	-0.02 (-0.20, 0.17)
DBP, 80 mmHg	0.57 (0.50, 0.65)	0.52 (0.05, 1.00)	-0.06 (-0.29, 0.17)	0.58 (0.50, 0.65)	0.77 (0.07, 1.48)	0.07 (-0.15, 0.30)
DBP, 90 mmHg	0.58 (0.46, 0.69)	0.46 (-0.25, 1.16)	-0.04 (-0.40, 0.32)	0.58 (0.47, 0.70)	0.68 (-0.36, 1.72)	0.16 (-0.19, 0.52)
DBP, 100 mmHg	0.58 (0.42, 0.74)	0.39 (-0.61, 1.40)	-0.02 (-0.53, 0.50)	0.59 (0.43, 0.75)	0.58 (-0.91, 2.07)	0.25 (-0.26, 0.77)

Table S2. Subgroup analyses based on participant characteristics, elevated LDL-C model; BMI, body mass index; cm, centimeters; CI, confidence interval; DBP, diastolic blood pressure; ISCO, International Standard Classification of Occupations (ISCO); kcal, kilocalories per day; kg/m², kilogram per square meters; mg/dL, milligram per deciliters; mmHg, millimeters of mercury; non-HDL-C, non-high-density lipoprotein cholesterol;

	Elevated non-HDL-C model

	Before update			After recalibration of c-slope and CITL		
**Subgroup analyses**	**AuROC (95%CI)**	**C-slope (95%CI)**	**CITL (95%CI)**	**AuROC (95%CI)**	**C-slope (95%CI)**	**CITL (95%CI)**
Gender						
Female subgroup	0.65 (0.61, 0.70)	0.83 (0.55, 1.12)	0.19 (0.02, 0.37)	0.65 (0.61, 0.70)	1.14 (0.75, 1.53)	0.03 (-0.14, 0.21)
Male subgroup	0.60 (0.52, 0.69)	0.73 (0.27, 1.20)	-0.23 (-0.50, 0.04)	0.61 (0.53, 0.69)	1.00 (0.36, 1.64)	-0.07 (-0.34, 0.19)
**ISCO code**						
ISCO code 2 subgroup	0.69 (0.63, 0.75)	1.01 (0.71, 1.30)	0.17 (-0.04, 0.38)	0.69 (0.63, 0.75)	1.38 (0.97, 1.78)	0.05 (-0.16, 0.25)
ISCO code 3 subgroup	0.63 (0.55, 0.71)	0.51 (0.16, 0.86)	0.04 (-0.27, 0.35)	0.63 (0.55, 0.71)	0.70 (0.22, 1.18)	-0.02 (-0.32, 0.29)
ISCO code 4 subgroup	0.62 (0.50, 0.75)	0.60 (0.05, 1.16)	0.13 (-0.31, 0.57)	0.62 (0.50, 0.75)	0.83 (0.06, 1.59)	0.09 (-0.34, 0.52)
ISCO code 9 subgroup	0.64 (0.46, 0.83)	1.00 (0.29, 1.71)	-0.50 (-0.96, -0.04)	0.65 (0.53, 0.77)	1.37 (0.40, 2.34)	-0.36 (-0.81, 0.09)
Other ISCO subgroup (1,5,6,7)	0.65 (0.53, 0.78)	0.52 (-0.29, 1.32)	0.19 (-0.46, 0.85)	0.64 (0.46, 0.83)	0.71 (-0.39, 1.81)	0.22 (-0.42, 0.85)
**BMI**						
BMI, 15 kg/m²	0.51 (0.41, 0.61)	0.95 (0.54, 1.37)	0.34 (0.02, 0.66)	0.53 (0.43, 0.63)	1.30 (0.74, 1.87)	-0.03 (-0.35, 0.28)
BMI, 20 kg/m²	0.55 (0.49, 0.60)	0.80 (0.50, 1.10)	0.19 (-0.01, 0.38)	0.56 (0.50, 0.62)	1.09 (0.68, 1.51)	-0.02 (-0.20, 0.17)
BMI, 25 kg/m²	0.58 (0.54, 0.63)	0.65 (0.37, 0.92)	0.03 (-0.12, 0.18)	0.59 (0.54, 0.64)	0.88 (0.51, 1.26)	0.00 (-0.15, 0.15)
BMI, 30 kg/m²	0.62 (0.53, 0.71)	0.49 (0.14, 0.85)	-0.12 (-0.36, 0.12)	0.62 (0.54, 0.71)	0.67 (0.19, 1.16)	0.02 (-0.21, 0.26)
BMI, 35 kg/m²	0.66 (0.53, 0.78)	0.34 (-0.16, 0.84)	-0.28 (-0.66, 0.10)	0.66 (0.53, 0.78)	0.47 (-0.21, 1.14)	0.04 (-0.33, 0.41)
BMI, 40 kg/m²	0.69 (0.52, 0.86)	0.19 (-0.47, 0.84)	-0.43 (-0.96, 0.09)	0.69 (0.52, 0.85)	0.26 (-0.64, 1.15)	0.06 (-0.45, 0.58)
**Age**						
Age, 20 years old	0.60 (0.50, 0.69)	0.49 (0.02, 0.97)	0.05 (-0.33, 0.43)	0.60 (0.50, 0.69)	0.68 (0.03, 1.32)	-0.18 (-0.55, 0.19)
Age, 25 years old	0.62 (0.57, 0.68)	0.58 (0.28, 0.89)	0.06 (-0.19, 0.30)	0.62 (0.57, 0.68)	0.80 (0.38, 1.22)	-0.10 (-0.33, 0.14)
Age, 30 years old	0.65 (0.61, 0.69)	0.67 (0.47, 0.87)	0.06 (-0.09, 0.21)	0.65 (0.61, 0.69)	0.92 (0.65, 1.19)	-0.02 (-0.17, 0.13)
Age, 35 years old	0.68 (0.62, 0.73)	0.76 (0.52, 1.00)	0.06 (-0.13, 0.26)	0.68 (0.62, 0.73)	1.04 (0.71, 1.37)	0.07 (-0.13, 0.26)
Age, 40 years old	0.70 (0.61, 0.79)	0.85 (0.46, 1.24)	0.07 (-0.26, 0.39)	0.70 (0.62, 0.79)	1.16 (0.63, 1.69)	0.15 (-0.17, 0.46)
**Fat percentage**						
Fat percentage, 10%	0.65 (0.57, 0.73)	1.25 (0.71, 1.79)	0.04 (-0.30, 0.37)	0.65 (0.57, 0.73)	1.71 (0.97, 2.44)	-0.11 (-0.44, 0.22)
Fat percentage, 20%	0.65 (0.60, 0.70)	0.93 (0.65, 1.21)	0.05 (-0.15, 0.25)	0.65 (0.60, 0.70)	1.27 (0.89, 1.65)	-0.05 (-0.25, 0.15)
Fat percentage, 30%	0.65 (0.61, 0.69)	0.61 (0.40, 0.82)	0.06 (-0.09, 0.22)	0.65 (0.62, 0.69)	0.83 (0.55, 1.12)	0.01 (-0.14, 0.16)
Fat percentage, 40%	0.65 (0.60, 0.71)	0.29 (-0.14, 0.73)	0.08 (-0.17, 0.33)	0.65 (0.60, 0.70)	0.40 (-0.20, 1.00)	0.08 (-0.17, 0.32)
Fat percentage, 50%	0.65 (0.57, 0.74)	-0.03 (-0.75, 0.69)	0.09 (-0.30, 0.48)	0.65 (0.57, 0.73)	-0.04 (-1.02, 0.95)	0.14 (-0.25, 0.53)
**Muscle percentage**						
Muscle percentage, 50%	0.65 (0.58, 0.72)	0.05 (-0.58, 0.68)	0.09 (-0.26, 0.43)	0.66 (0.59, 0.72)	0.07 (-0.79, 0.93)	0.11 (-0.23, 0.45)

Muscle percentage, 60%	0.65 (0.61, 0.70)	0.40 (0.05, 0.74)	0.07 (-0.13, 0.27)	0.66 (0.61, 0.70)	0.54 (0.08, 1.01)	0.05 (-0.15, 0.25)
Muscle percentage, 70%	0.65 (0.61, 0.69)	0.74 (0.56, 0.93)	0.06 (-0.10, 0.21)	0.66 (0.62, 0.69)	1.02 (0.76, 1.27)	-0.01 (-0.16, 0.14)
Muscle percentage, 80%	0.65 (0.59, 0.72)	1.09 (0.70, 1.48)	0.04 (-0.21, 0.30)	0.65 (0.59, 0.72)	1.49 (0.95, 2.02)	-0.07 (-0.33, 0.18)
Muscle percentage, 90%	0.65 (0.56, 0.75)	1.44 (0.75, 2.12)	0.03 (-0.37, 0.43)	0.65 (0.56, 0.75)	1.96 (1.03, 2.90)	-0.14 (-0.54, 0.27)
**Waist-to-height ratio**						
Waist-to-height ratio, 0.4	0.50 (0.43, 0.58)	0.85 (0.49, 1.21)	0.29 (0.03, 0.55)	0.51 (0.44, 0.59)	1.17 (0.68, 1.66)	-0.02 (-0.27, 0.24)
Waist-to-height ratio, 0.5	0.58 (0.54, 0.63)	0.70 (0.42, 0.98)	0.07 (-0.08, 0.22)	0.59 (0.54, 0.63)	0.96 (0.58, 1.35)	0.00 (-0.15, 0.15)
Waist-to-height ratio, 0.6	0.66 (0.58, 0.73)	0.55 (0.20, 0.91)	-0.15 (-0.40, 0.10)	0.66 (0.58, 0.73)	0.76 (0.27, 1.25)	0.02 (-0.23, 0.26)
Waist-to-height ratio, 0.7	0.73 (0.61, 0.84)	0.40 (-0.12, 0.93)	-0.37 (-0.80, 0.07)	0.72 (0.60, 0.84)	0.55 (-0.16, 1.27)	0.03 (-0.39, 0.46)
Waist-to-height ratio, 0.8	0.79 (0.64, 0.93)	0.25 (-0.47, 0.98)	-0.58 (-1.22, 0.05)	0.78 (0.63, 0.93)	0.35 (-0.64, 1.34)	0.05 (-0.57, 0.67)
**Metabolic age**						
Metabolic age, 20 years	0.50 (0.43, 0.58)	0.80 (0.50, 1.10)	0.15 (-0.06, 0.37)	0.56 (0.49, 0.62)	1.09 (0.68, 1.51)	-0.05 (-0.26, 0.16)
Metabolic age, 30 years	0.58 (0.54, 0.63)	0.69 (0.45, 0.93)	0.09 (-0.07, 0.24)	0.57 (0.52, 0.62)	0.95 (0.62, 1.28)	-0.01 (-0.17, 0.14)
Metabolic age, 40 years	0.66 (0.58, 0.73)	0.58 (0.33, 0.84)	0.02 (-0.14, 0.18)	0.58 (0.52, 0.64)	0.80 (0.45, 1.15)	0.02 (-0.14, 0.19)
Metabolic age, 50 years	0.73 (0.61, 0.84)	0.48 (0.14, 0.82)	-0.05 (-0.28, 0.19)	0.60 (0.51, 0.69)	0.65 (0.19, 1.12)	0.06 (-0.17, 0.29)
Metabolic age, 60 years	0.79 (0.64, 0.93)	0.37 (-0.09, 0.82)	-0.11 (-0.44, 0.22)	0.61 (0.49, 0.73)	0.50 (-0.12, 1.13)	0.10 (-0.22, 0.42)
**DBP**						
DBP, 50 mmHg	0.61 (0.52, 0.69)	0.76 (0.34, 1.19)	0.31 (-0.04, 0.66)	0.62 (0.53, 0.70)	1.04 (0.46, 1.62)	-0.02 (-0.36, 0.32)
DBP, 60 mmHg	0.62 (0.56, 0.68)	0.75 (0.44, 1.05)	0.20 (-0.03, 0.42)	0.63 (0.57, 0.68)	1.02 (0.61, 1.43)	-0.01 (-0.23, 0.21)
DBP, 70 mmHg	0.63 (0.59, 0.68)	0.73 (0.50, 0.95)	0.09 (-0.07, 0.24)	0.64 (0.60, 0.68)	1.00 (0.69, 1.30)	0.00 (-0.15, 0.15)
DBP, 80 mmHg	0.65 (0.59, 0.70)	0.71 (0.47, 0.95)	-0.03 (-0.22, 0.16)	0.65 (0.59, 0.70)	0.97 (0.64, 1.30)	0.01 (-0.18, 0.19)
DBP, 90 mmHg	0.66 (0.58, 0.75)	0.69 (0.35, 1.03)	-0.14 (-0.44, 0.16)	0.66 (0.57, 0.74)	0.95 (0.48, 1.41)	0.01 (-0.28, 0.31)
DBP, 100 mmHg	0.68 (0.56, 0.79)	0.68 (0.21, 1.15)	-0.25 (-0.68, 0.18)	0.67 (0.55, 0.78)	0.93 (0.28, 1.57)	0.02 (-0.40, 0.44)
